# A Clinical Lecture on Goitre

**Published:** 1898-06-11

**Authors:** J. Lacy Firth

**Affiliations:** AssistantSurgeon to the Bristol General Hospital


### Medical Progress and Hospital Clinics.

[*The Editor will be glad to receive offers of co-operation and contributions from members of the profession. All letters should be addressed to* The Editor, at the Office, 28 & 29, Southampton Street, Strand, London, W.C.]

### A CLINICAL LECTURE ON GOITRE.

By J. Lacy Firth, M.D., M.S., F.R.C.S., Assistant-Surgeon to the Bristol General Hospital.

(*Concluded from page* 167.)

Bearing in mind the risks of operations on the thyroid, to which I shall allude again in a moment, you should not recommend an operation unless the inconvenience suffered is sufficiently great to justify exposing your patient to them in the search for relief.

Then we come to the important group of cases in which there is dyspnœa. For these cases operation, and early operation, is usually indicated. In the mildest cases of dyspnœa a trial of the iodine treatment may first be made, but the danger of an attack of sudden and fatal dyspnœa must ever be borne in mind, and if improvement does not quickly follow that treatment it should be discarded, and an operation performed.

The operations performed upon the thyroid have for their object the immediate diminution of the size of the gland and the prevention of the future enlargement of the portion left. In the special case of malignant disease the object is to completely remove the gland, but this is not to be thought of for other diseases, for we have seen above that complete removal of the gland is very likely to produce the serious condition of cachexia strumipriva, allied to myxœdema. A very small portion of gland left behind seems, however, sufficient to guard against this evil.

The operations which may be performed for the reduction in size of the gland are of three grades of simplicity. Placed in the order of their simplicity, they are: (1) Enucleation (Socin); (2) division or excision of the thyroid isthmus; and (3) excision of a lobe, with or without the isthmus (Kocher).

Some other operations have been successfully undertaken for the same purposes, such as ligature of the superior and inferior thyroid arteries, scraping out of the gland after an incision into it, and ligature of the cervical sympathetic trunk, but those given in the above list are the best for ordinary cases.

It is more in the treatment of exophthalmic goitre that ligature of the arteries has been done, and only for that disease has the cervical sympathetic been divided. These methods have been tried because in that disease thyroidectomy has proved specially dangerous.

The operation of enucleation is only adapted for goitres of the adenomatous type, and when also the desired reduction in size of the goitre can be produced by the removal of a few encapsuled adenomata. When the adenomata are numerous and diffused thyroidectomy should be the operation selected. Of the two forms of thyroidectomy in our list it may be said that the removal of a lobe is more radical and more certain in its results, but on the other hand is more dangerous from hæmorrhage, from the liability to injury of the recurrent laryngeal nerve, and from septic cellulitis. Excision of the isthmus is a comparatively simple operation, unless its size is very excessive, and yet is of well-established efficiency, and should therefore be the operation selected in the first instance in most cases. If, however, it be important in any case to obtain very quickly a relief of pressure on the trachea, if the enlargement is chiefly of the lateral lobes, the isthmus being comparatively small, and that enlargement be not colossal, or if the isthmus have been excised at a previous operation which has failed to procure relief, then you would act wisely in removing one of the affected lobes. The operation becomes more difficult and dangerous when the thyroid enlargement is partially substernal. When, therefore, there are indications that the enlargement is taking that direction, thyroidectomy should be performed early, if the growth of the gland cannot be otherwise checked.

The treatment of solitary cysts of the thyroid should be by excision if possible, otherwise by incision and drainage. If it is important to avoid a scar, there remains the treatment by injection of the cyst, if solitary and presumably encapsuled, with iodine or perchloride of iron solution, leaving in the cannula until suppuration is established, and then substituting a small drainage tube. The responsibility of starting a suppurative process in the neck, however, cannot be undertaken without anxiety, and I doubt if many surgeons nowadays would do it.

The surgical treatment of exophthalmic goitre may be justified if all other remedies have failed, and the patient appears otherwise condemned to years or to a lifetime of invalidism, for it has met with a certain amount of success. Of course, if there are serious pressure symptoms the case is different, and operation should in that case be undertaken without hesitation. The special risks of thyroidectomy in exophthalmic goitre are due to the facts that the patients bear the operation badly, and may sink under it from shock; that the capsule of the gland is specially vascular, increasing the risk of hæmorrhage; and that there is a liability, in the 48 hours immediately following the operation, to the onset of a peculiar and dangerous train of symptoms often ending in death. These symptoms are a rapid rise of temperature and pulse rate, albuminuria, delirium, and coma. These symptoms are believed to be due to an excessive absorption of thyroid secretion from the gland, either from the section surfaces or other parts. Perhaps there is an excessive secretion as a result of the free handling of the gland. The only precautions that can be taken to guard against this complication are the avoidance of much manipulation of the gland during the operation, and the establishment of free drainage at its conclusion. The treatment of malignant disease of the thyroid, whether carcinomatous or sarcomatous, is extremely unsatisfactory. The disease can rarely be recognised before it is too late to hope for a radical cure by operation. Early in their evolution these growths tend to spread between and around the œsophagus and trachea, and between the large vessels of the neck, rendering their removal hazardous and usually incomplete. It follows that it is rarely justifiable to operate for such cases.

